# In Vitro Effects of Inulin, Resistant Dextrin, and Stachyose on the Functional Properties and Digestibility of Corn Starch

**DOI:** 10.1002/fsn3.70165

**Published:** 2025-09-18

**Authors:** Mengli Yao, Xiaofeng Han, Nick Pi, Ruoyong Wang, Zhong Han, Hua Dong, Zhiqiao Zhou, Jia Liu, Xue Wang, Shenglin Duan, Peng Yuan, Jie Liu

**Affiliations:** ^1^ Beijing Key Laboratory of the Innovative Development of Functional Staple and Nutritional Intervention for Chronic Disease China National Research Institute of Food and Fermentation Industries Co., LTD Beijing China; ^2^ Nansha College Preparatory Academy Guangzhou China; ^3^ Air Force Characteristic Medical Center Beijing China; ^4^ School of Food Science and Engineering South China University of Technology Guangzhou China; ^5^ School of Food and Biology Changchun Vocational and Technical College Jilin China; ^6^ Heilongjiang Feihe Dairy co. ltd. Beijing China; ^7^ Key Laboratory of Geriatric Nutrition and Health Beijing Technology and Business University, Ministry of Education Beijing China

**Keywords:** enzyme inhibition, glucose adsorption, inulin, pasting, resistant dextrin, stachyose

## Abstract

This study explores the effects of three soluble dietary fibers (SDFs)—inulin, resistant dextrin, and stachyose—on the in vitro digestion of corn starch. Incorporation of SDFs significantly influences starch physicochemical properties by reducing swelling power, solubility, pasting viscosity, and breakdown, while increasing pasting temperature and particle size (*p* < 0.05). During in vitro digestion, the presence of SDFs significantly decreased the rapidly digestible starch (RDS) content and increased the resistant starch (RS) content (*p* < 0.05). Microscopic observations reveal that SDFs alter the microstructure of starch gels, enhancing the stability of the gel matrix. Fourier‐transform infrared (FT‐IR) spectroscopy suggests the formation of hydrogen bonds between SDFs and starch. Furthermore, SDFs exhibit glucose adsorption capacity and α‐amylase inhibitory activity, highlighting their multiple mechanisms in modulating starch digestion. The differential effects of SDFs depend on their molecular structure and physicochemical properties, such as chain length and hydroxyl group density. These findings offer new insights into the role of SDFs in modulating starch digestion and highlight their potential applications in designing low‐glycemic and fiber‐enriched functional foods.

## Graphical abstract

This study investigates the effects of inulin, resistant dextrin, and stachyose on the in vitro digestion, pasting properties, and structural characteristics of corn starch. Soluble dietary fibers (SDFs) were found to reduce starch digestibility, enhance resistant starch content, and modify starch microstructure through hydrogen bonding. Furthermore, SDFs exhibited notable glucose adsorption capacity and enzyme inhibition effects, offering valuable insights for developing low‐glycemic and fiber‐enriched functional foods.

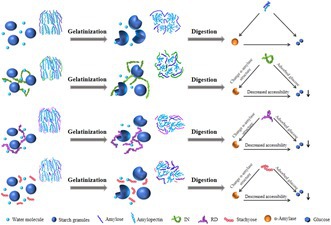

## 1 Introduction

In recent years, the addition of non‐starch components to food systems has gained significant attention as an effective strategy for reducing the glycemic index (GI) of starch‐based foods (Qiu et al. 2016). Recent studies have demonstrated that soluble dietary fibers (SDFs) can modulate the pasting properties, retrogradation characteristics, and digestive kinetics of starch, thereby delaying postprandial blood glucose spikes (Zhang et al. 2021). Furthermore, SDFs from different sources exhibit distinct structural and functional properties (Xiong et al. 2024). For example, Zou et al. (2022) systematically compared SDFs extracted from licorice, apple, chicory, and flaxseed, revealing notable differences in water‐holding capacity, swelling power, and α‐amylase inhibitory activity.

Building on this, the present study focuses on three widely studied SDFs with distinct molecular structures: inulin (IN), resistant dextrin (RD), and stachyose (Sta). Each of these SDFs exhibits unique structural and functional properties, making them promising candidates for modulating starch digestion. IN, a naturally occurring linear fructan found in plants such as Jerusalem artichoke, chicory, and agave, is composed of D‐fructofuranose units linked by β‐2,1 bonds (Luo et al. 2017). It has been widely recognized for its health benefits, including regulating gut microbiota, improving blood glucose levels, and enhancing mineral absorption (Ji, Yang, et al. 2021; Ji, Yin, et al. 2021). RD, derived from starch through enzymatic hydrolysis or acid‐heat treatment, possesses a branched structure with diverse glycosidic linkages, including α‐1,2, α‐1,3, β‐1,2, and β‐1,6 glycosidic linkages (Qin et al. 2020). These unique linkages make it resistant to digestion in the upper gastrointestinal tract, allowing it to modulate postprandial satiety and glycemic responses (Yu et al. 2022). Similarly, Sta, a tetrasaccharide commonly found in plants of the Leguminosae, Lamiaceae, and Scrophulariaceae families, consists of two α‐galactose, one α‐glucose, and one β‐fructose moiety. Its distinct α‐1,6 and α‐1,2 glycosidic bonds prevent digestion in the small intestine, enabling its prebiotic activity in the large intestine. Research has highlighted Sta's roles in alleviating constipation, enhancing immunity, and reducing blood glucose levels (Xu et al. 2024).

While IN has been extensively studied for its effects on starch digestibility and structural modifications, research on RD and Sta remains limited (Ji, Yang, et al. 2021; Ji, Yin, et al. 2021; Luo et al. 2017; Witczak et al. 2014). Current reports primarily focus on IN's impact on altering starch crystallinity, gelation behavior, and digestion performance, with little emphasis on the comparative effects of RD and Sta. For example, Ji, Yang, et al. (2021) and Ji, Yin, et al. (2021) demonstrated how variations in IN molecular weight influence starch digestion and gelation. However, there are no systematic studies that investigate the inhibition of starch hydrolysis efficiency and hypoglycemic mechanisms of RD and Sta. Figure S1 depicts the distinct structural characteristics of IN, RD, and Sta, highlighting their molecular differences that potentially influence starch gelatinization, digestion, and interaction mechanisms.

Based on previous laboratory findings, it has been established that all three SDFs—IN, RD, and Sta—can inhibit starch hydrolysis to varying extents (Figure S2). Therefore, this study aims to systematically compare the effects of these SDFs with different molecular weights on the physicochemical properties, gelatinization, structural characteristics, and in vitro digestibility of corn starch (CS). The results provide valuable insights into the distinct mechanisms by which SDFs influence starch digestion and offer a foundation for applying RD, Sta, IN, and other similar dietary fibers in the formulation of starch‐based functional foods.

## 2 Materials and Methods

### 2.1 Materials and Reagents

Resistant dextrin (degree of polymerization: 2–10, molecular weight: 1600–2000 Da) was purchased from ADM Japan Ltd. (Tokyo, Japan). Inulin (degree of polymerization: 2–60, average molecular weight: 6179.36 Da) was obtained from Shanghai Shanyi Biological Technology Co. Ltd. (Shanghai, China), and stachyose (degree of polymerization: 4, molecular weight: 666.59 Da) was provided by Beijing Xinshengyuan Biomedical Technology Co. Ltd. (Beijing, China). Corn starch (CS), containing 30.9% amylose and 51.4% moisture, was purchased from Xuzhou Lvran Foodstuff Co. Ltd. (Jiangsu, China).

α‐Amylase (from porcine pancreas, 10.2 U mg^−1^) provided by Sigma‐Aldrich (St. Louis, MO, USA). α‐Glucosidase (from *Saccharomyces cerevisiae* , 32.2 U mg^−1^) purchased from Yuanye Biotech (Shanghai, China). Pepsin (from porcine gastric mucosa, ≥ 250 U mg^−1^), pancreatin (from porcine pancreas, ≥ 125 U mg^−1^), and amyloglucosidase (from Aspergillus niger, 142 U mg^−1^) were all provided by Sigma‐Aldrich (St. Louis, MO, USA). Bile salts (No. 3) were obtained from Macklin Biotech (Shanghai, China). Glucose oxidase‐peroxidase (GOPOD) assay kits were purchased from Megazyme International Ireland Ltd. (Wicklow, Ireland). All other chemicals used were of analytical grade unless otherwise specified.

### 2.2 Swelling Power and Solubility

SDFs were weighed to constitute 5%, 10%, 15%, 20%, and 25% (w/w) of the corn CS mass. The fibers were transferred into 2 mL centrifuge tubes, and 1 mL of distilled water was added to each tube. The mixtures were vortexed or shaken vigorously until complete dissolution was achieved. Subsequently, 40 mg of CS was added to each tube, and the mixtures were thoroughly mixed to ensure uniform dispersion. The samples were heated in a water bath at 95°C for 30 min with intermittent shaking to prevent sedimentation. After heating, the tubes were cooled to room temperature and centrifuged at 4472 rpm for 20 min to separate the precipitate and supernatant. The precipitate was weighed, and the supernatant was carefully transferred to a drying dish. The supernatant was dried at 100°C until a constant weight was achieved. The dried mass was recorded, and swelling power and solubility were calculated using the method described by Xie et al. (2024),

(1) $$S\left(\%\right)=\frac{W_{2}}{W_{1}}\times 100$$

(2) $$\mathrm{SP}\left(\%\right)=\frac{W_{3}}{W_{1}-\left(100-S\right)}\times 100$$

where *W*_1_ is the dry weight of the sample (mg), *W*_2_ is the weight of the dried supernatant (mg), and *W*_3_ is the weight of the precipitate (mg).

### 2.3 Pasting Properties

SDFs effects on starch pasting characteristics were assessed using a Rapid Visco Analyzer (RVA, 3‐D, Newport Scientific). Mixed powders were prepared by incorporating 5%, 10%, 15%, and 20% (w/w) of IN, RD, and Sta based on the starch mass. Accurately weigh 3.0 g of each mixed powder into an aluminum canister. Add 25 mL of distilled water to the canister and stir thoroughly to ensure uniform mixing. Set the instrument stirring speed to 960 rpm for the first 10 s, followed by a reduction to 160 rpm. The slurry was maintained at 50°C for 1 min, then heated to 95°C at a controlled rate of 5.4°C min^−1^. After holding at 95°C for 5 min, the sample was cooled to 50°C at the same rate and held for 2 min. Pasting parameters, including peak viscosity, breakdown viscosity, and setback viscosity, were continuously recorded throughout the test (Zheng, Huang, et al. 2021; Zheng, Wang, et al. 2021).

### 2.4 Particle Size Distribution

The particle size distribution of the samples was determined using a laser diffraction particle size analyzer (LS13320, Beckman Coulter, USA). The paste was immediately transferred from the RVA to the sample dispersion system of the analyzer. An appropriate amount of sample was added to achieve the recommended obscuration range (10%–20%), as suggested by the manufacturer. Care was taken to ensure the sample was evenly dispersed in the liquid medium without any aggregation.

### 2.5 Digestibility Characteristics

With a few minor modifications, the in vitro digesting system was developed in accordance with Wang et al. (2023). The pastes obtained from the RVA were freeze‐dried and ground into uniform fine powders for subsequent in vitro digestion experiments. Simulated in vivo digestion conditions were replicated using a dissolution tester (RC‐806; Beijing Zhongxi Huada Technology Co., China) set at 37°C and 190 rpm. Accurately, 1.0 g of sample (on a dry starch basis) was weighed and mixed with 16 mL of simulated gastric fluid (containing porcine pepsin at 2000 U mL^−1^ in 2 M sodium acetate buffer, pH 5.2). The pH was adjusted to 2.0 using 1 M HCl, and the mixture was homogenized by shaking. Digestion was performed using the dissolution tester at 37°C and 190 rpm for 1 h. Subsequently, the pH was adjusted to 7.0 using 1 M NaOH, followed by the addition of 18 mL of simulated intestinal fluid (containing porcine pancreatin at 800 U mL^−1^, bile salts at 10 mM, and CaCl_2_ at 0.3 mM). The mixture was incubated at 37°C and 130 rpm for 120 min.

At 0, 20, 30, 60, 90, and 120 min of intestinal digestion, 500 μL of digestion fluid was withdrawn and immediately subjected to enzyme inactivation by boiling for 10 min. The inactivated fluid was centrifuged at 1118 rpm for 5 min, and 100 μL of the supernatant was mixed with 900 μL of amyloglucosidase solution (400 U mL^−1^). The mixture was incubated at 37°C for 10 min, followed by a second boiling step for 10 min to inactivate the enzymes. The glucose content in the final sample was determined by measuring the absorbance at 505 nm using a GOPOD assay kit. The starch content was calculated by multiplying the glucose content by 0.9 (Englyst et al. 1992; Zheng, Huang, et al. 2021; Zheng, Wang, et al. 2021):

(3) $$\mathrm{RDS}\left(\%\right)=\left(G_{20}-\mathrm{FG}\right)\times 0.9\times 100/\mathrm{TG}$$

(4) $$\mathrm{SDS}\left(\%\right)=\left(G_{120}-G_{20}\right)\times 0.9\times 100/\mathrm{TG}$$

(5) $$\mathrm{RS}\left(\%\right)=\left(1-\mathrm{RSD}-\mathrm{SDS}\right)\times 100$$

where *G*_20_ and *G*_120_ represent the glucose content (mg) at 20 min and 120 min, respectively. FG represents the free glucose content (mg) at 0 min. TG represents the total starch mass (mg).

### 2.6 Scanning Electron Microscopy (SEM)

The freeze‐dried samples obtained from the RVA were cut into thin slices using a utility knife. The slices were mounted onto a sample holder with double‐sided conductive tape to ensure proper adhesion. The morphological characteristics of the samples were observed under a scanning electron microscope (SEM, Phenom Pro X, Thermo Fisher Scientific, USA) at an acceleration voltage of 10 kV and a magnification of 300×. All observations were conducted in high vacuum mode to ensure image clarity (Zheng, Huang, et al. 2021; Zheng, Wang, et al. 2021).

### 2.7 Fourier‐Transform Infrared (FT‐IR) Spectroscopy

The starch pastes were analyzed using an FT‐IR spectrometer (Spectrum 100, Perkin Elmer Co., USA). The pastes obtained from the RVA were freeze‐dried, and 0.01 g of the dried sample was thoroughly mixed with 1.00 g of KBr powder to form a pellet for measurement. Spectral data were collected over the range of 400–4000 cm^−1^ with a resolution of 4 cm^−1^ and an average of 64 scans per sample. The absorbance ratio at 1047 cm^−1^ to 1022 cm^−1^ (*R*_1047/1022_) and the absorbance ratio at 995 cm^−1^ to 1022 cm^−1^ (*R*_995/1022_) were calculated to evaluate structural changes in the starch samples (Lan et al. 2024).

### 2.8 Adsorption of Glucose by SDFs

#### 2.8.1 Glucose Adsorption Capacity (GAC)

To determine the glucose adsorption capacity of SDFs, glucose solutions with concentrations of 2, 4, 6, and 8 mg mL^−1^ were prepared. Then, 10 mL of each glucose solution was mixed with 0.1 g of different SDFs samples. The mixtures were thoroughly mixed and incubated in a water bath at 37°C for 6 h. After incubation, the samples were centrifuged at 1610 rpm for 20 min, and the glucose content in the supernatant was determined by measuring absorbance at 505 nm using a GOPOD assay kit (Tang et al. 2022). Deionized water was used as a negative control, and a solution without SDFs was used as a positive control. The glucose adsorption capacity was calculated as follows (Equation 6):

(6) $$\mathrm{GAC}\left(\frac{\mathrm{mg}}{g}\right)=\frac{C_{0}-C_{e}}{W}$$

where *C*_0_ is the initial glucose concentration in the solution (mg mL^−1^). *C*_e_ is the equilibrium glucose concentration in the solution (mg mL^−1^). W is the weight of the SDFs samples (g).

#### 2.8.2 Morphological Characteristics of SDFs by Electron Microscopy

The particle morphology of the samples was observed at an accelerating voltage of 5.0 kV and magnifications of 250× and 1000× using a scanning electron microscope (SEM, Phenom Pro X, Thermo Fisher Scientific, USA) operated in high vacuum mode and equipped with a secondary electron detector.

### 2.9 Interaction of Three Types of SDFs With α‐Amylase

#### 2.9.1 α‐Amylase Inhibtion

The α‐amylase inhibitory activity was evaluated following the method of Wang et al. (2023) with slight modifications. Briefly, 200 μL of SDFs solutions at varying concentrations (40 mg mL^−1^, 60 mg mL^−1^, 80 mg mL^−1^, 100 mg mL^−1^, 120 mg mL^−1^, and 140 mg mL^−1^) were mixed with α‐amylase‐phosphate buffer (5 U mL^−1^) and incubated at 37°C in a water bath shaker for 10 min. Subsequently, 200 μL of 1% pre‐gelatinized starch solution was added, and the mixture was incubated for an additional 10 min under the same conditions. The reaction was terminated by adding 400 μL of dinitrosalicylic acid (DNS) reagent, followed by heating in a boiling water bath for 10 min. The reduction of 3‐dinitrosalicylic acid to a reddish‐brown compound by reducing sugars produced an absorption peak at 540 nm. The α‐amylase activity was determined by measuring the absorbance at 540 nm, and a blank or control (buffer replacing SDFs solutions or enzyme) was included. The α‐amylase inhibitory activity was calculated using the following equation (Equation 7), and univariate nonlinear regression analysis was performed based on the concentration and inhibition rate, and the IC_50_ values of IN, RD, and Sta were calculated by performing nonlinear regression analysis of the concentration and inhibition rate using GraphPad Prism 9.0,

(7) $$\text{Inhibitory activity}\left(\%\right)=\left(1-\frac{{\mathrm{OD}}_{A}-{\mathrm{OD}}_{a}}{{\mathrm{OD}}_{B}-{\mathrm{OD}}_{b}}\right)\times 100$$

where OD_A_ is the absorbance value of the experimental group, and OD_a_ is the blank absorbance value of the experimental group. OD_B_ is the absorbance value of the control group, and OD_b_ is the blank absorbance value of the control group.

#### 2.9.2 Fluorescence Quenching

The fluorescence spectra of α‐amylase under different SDFs concentrations were collected to analyze the binding interactions between SDFs and α‐amylase, following the method described by Zheng, Shi, et al. (2020) and Zheng, Yang, et al. (2020) with some modifications. Briefly, a mixture containing 0.3 mL of SDFs and 3.0 mL of α‐amylase solution (5 U mL^−1^) was incubated at three different temperatures (298 K, 304 K, and 310 K) for 15 min. A control was prepared by replacing the SDFs with an equal volume (0.3 mL) of buffer solution. Fluorescence emission spectra of all solutions were recorded with an excitation wavelength of 280 nm and an emission wavelength range of 300–500 nm. Background values were subtracted from the emission spectra of the corresponding SDFs solutions and buffer. The Stern‐Volmer equation was applied to determine the type of quenching induced by the quencher (Equation 8):

(8) $$F_{c}=F_{m}e^{\left(A_{1}+A_{2}\right)/2}$$

where *F*_c_ and *F*_m_ are the corrected and measured fluorescence intensities, respectively. *A*_1_ and *A*_2_ are the absorbances of the SDFs sample at the excitation and emission wavelengths, respectively.

To further investigate the quenching mechanism, the Stern–Volmer equation was used:

(9) $$\frac{F_{0}}{F}=1+K_{\mathrm{sv}}\times \left[Q\right]=1+K_{q}\times \tau_{0}\times \left[Q\right]$$

where *F*_0_ and *F* are the fluorescence emission intensities of *α*‐amylase in the absence and presence of SDFs, respectively; *K*_sv_ is the Stern–Volmer quenching constant; *Q* is the concentration of SDFs; *K*_q_ is the fluorescence quenching rate constant; τ_0_ is the lifetime of the fluorescence in the absence of the quencher, valued at 10^−8^ s.

### 2.10 Statistical Analysis

Experiments were conducted in triplicate. Data were plotted using GraphPad Prism 9. Results are presented as mean ± standard deviation (*x* ± *s*). Statistical significance was analyzed by one‐way ANOVA and Duncan's test using SPSS 24.0, and significance was established at *p* < 0.05.

## 3 Results and Discussion

### 3.1 Swelling Power and Solubility

Swelling power (SP) and solubility (S), key indicators of starch's water‐holding capacity, were utilized to evaluate differences among starch samples in this study (Zheng, You, et al. 2019; Zheng, Wang, et al. 2019). When starch molecules are heated in excess water, their crystalline structure is disrupted, resulting in increased swelling and solubility. Consequently, changes in S and SP during the gelatinization process of CS‐SDFs mixtures were analyzed to investigate the effects of SDFs on the structure and molecular transformations of CS.

**FIGURE 1:**
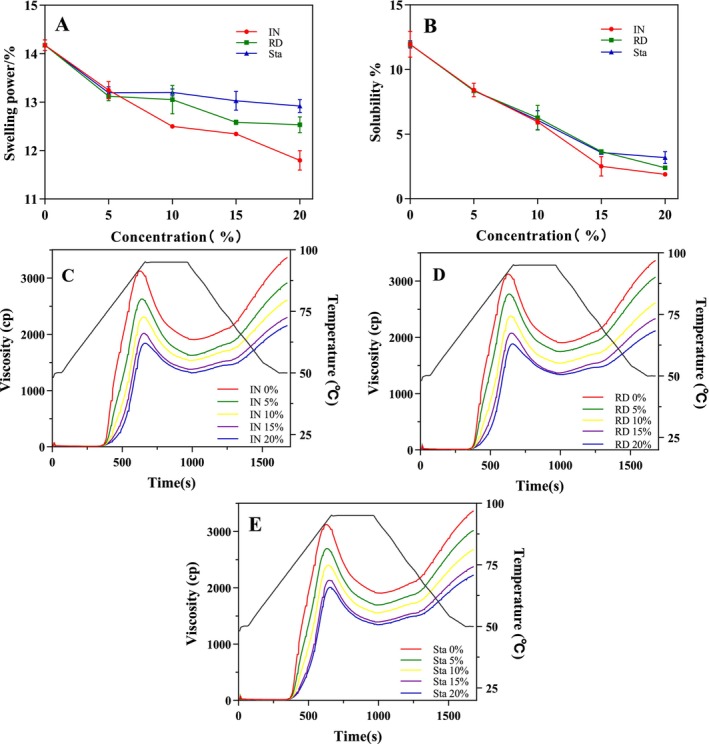
The effects of adding different amounts of SDFs on the swelling power (A), solubility (B), and pasting properties (C–E) of CS.

The addition of SDFs significantly reduced the SP and S of CS, and this effect became more pronounced with increasing amounts of SDFs (*p* < 0.05) (Figure 1A,B). Previous studies have demonstrated that non‐starch components can inhibit starch hydrolysis by reducing the swelling capacity of starch (Xie et al. 2024; Ye et al. 2018). The reduction in SP and S of starch may be attributed to two factors. On one hand, the interaction between SDF and amylose on the surface of starch granules may result in the formation of a protective film on the granule surface, thereby preventing further swelling and amylose leaching (Jia et al. 2023). On the other hand, the residual amylose within the starch granules may increase, enhancing the crystallinity and structural order within the granules, which further reduces starch swelling (Zheng, You, et al. 2019; Zheng, Wang, et al. 2019).

In addition, the effect of IN on starch was more pronounced compared to RD and Sta (Figure 1A,B). This could be attributed to the longer molecular chain structure of IN, which allows it to more effectively encapsulate the surface of starch molecules and inhibit starch swelling (Luo et al. 2017). Therefore, the incorporation of IN, RD, and Sta can reduce the SP and S of starch, with the extent of these effects varying depending on the molecular structure and properties of the SDFs.

### 3.2 Pasting Properties

Starch is insoluble in water at room temperature but gelatinizes when heated. During pasting, starch granules swell, crystalline regions are disrupted, amylose leaches out, and system viscosity increases (Gao et al. 2021; Wang et al. 2019). As shown in Figure 1C–E, the pasting curves of CS‐SDFs mixtures indicate that increasing SDFs concentrations reduce viscosity at all stages. Table 1 summarizes the pasting properties, showing that SDFs significantly lower peak viscosity (PV) and trough viscosity (TV) while raising pasting temperature (PT), with stronger effects at higher SDFs levels. These changes result from SDFs restricting water availability, reducing starch swelling and amylose leaching, and delaying pasting (Lutfi et al. 2017; Wang et al. 2019). Similar findings by Ma et al. (2019) further confirm that added polysaccharides reduce water availability for starch pasting through competitive water binding.

**TABLE 1:** Effect of different SDFs on pasting properties of CS.

Samples	PV(cP)	BV(cP)	BD(cP)	FV(cP)	SB(cP)	PT(°C)
CS	3117 ± 70^a^	1845 ± 61^a^	1272 ± 59^a^	3312 ± 77^a^	3277 ± 76^a^	73.4 ± 0.49^e^
CS‐5%IN	2623 ± 6^c^	1641 ± 21^c^	983 ± 26^b^	2905 ± 7^c^	1265 ± 11^e^	74.7 ± 0.00^d^
CS‐10%IN	2301 ± 2^e^	1530 ± 6^d^	771 ± 4^d^	2597 ± 13^e^	1067 ± 28^f^	76.0 ± 0.18^b^
CS‐15%IN	2018 ± 3^g^	1384 ± 9^ef^	635 ± 6^f^	2307 ± 14^fg^	924 ± 7^g^	77.6 ± 0.04^a^
CS‐20%IN	1866 ± 37^h^	1320 ± 10^g^	546 ± 28^g^	2132 ± 26^h^	812 ± 36^hi^	77.7 ± 0.00^a^
CS‐5%Sta	2759 ± 87^b^	1719 ± 35^b^	1040 ± 9^b^	3053 ± 53^b^	3024 ± 5^b^	74.2 ± 0.25^d^
CS‐10%Sta	2407 ± 11^d^	1559 ± 3^d^	848 ± 35^c^	2698 ± 33^d^	2676 ± 51^c^	75.3 ± 0.81^d^
CS‐15%Sta	2145 ± 15^f^	1409 ± 24^e^	736 ± 3^de^	2384 ± 16^f^	2362 ± 34^d^	77.2 ± 0.21^a^
CS‐20%Sta	2036 ± 45^g^	1352 ± 12^fg^	684 ± 33^ef^	2241 ± 30^g^	890 ± 18^gh^	77.5 ± 0.00^a^
CS‐5%Sta	2759 ± 87^b^	1719 ± 35^b^	1040 ± 9^b^	3053 ± 53^b^	3024 ± 5^b^	74.2 ± 0.25^d^
CS‐10%Sta	2407 ± 11^d^	1559 ± 3^d^	848 ± 35^c^	2698 ± 33^d^	2676 ± 51^c^	75.3 ± 0.81^d^
CS‐15%Sta	2145 ± 15^f^	1409 ± 24^e^	736 ± 3^de^	2384 ± 16^f^	2362 ± 34^d^	77.2 ± 0.21^a^
CS‐20%Sta	2036 ± 45^g^	1352 ± 12^fg^	684 ± 33^ef^	2241 ± 30^g^	890 ± 18^gh^	77.5 ± 0.00^a^

*Note:* Reported values correspond to the mean ± standard deviation. Different letters in the same column indicate significant differences (*p* < 0.05).

Abbreviations: BD, breakdown; FV, final viscosity; PT, pasting temperature; PV, peak viscosity; SB, setback; TV, trough viscosity.

In the RVA characteristic parameters, the breakdown value (BD) represents the starch granules' resistance to thinning during continuous heating and mechanical shearing. It is calculated as the difference between PV and TV, with higher BD values indicating less stable starch granules (Lutfi et al. 2017). The setback value (SB), on the other hand, reflects the retrogradation tendency of the starch paste during cooling, indicating amylose rearrangement and paste viscosity increase. As shown in Table 1, the BD of the samples decreased significantly with increasing SDFs content (*p* < 0.05), suggesting improved system stability. This is likely because SDFs molecules form coatings or aggregates on the surface of starch granules, enhancing resistance to heat and shear forces (Zhang et al. 2023). Reduced BD is also associated with decreased RDS content, indicating lower digestibility. Similarly, the SB decreased significantly with higher SDFs levels (*p* < 0.05), likely due to interactions between SDFs and leached amylose, which lower local amylose concentration and disrupt starch recrystallization, delaying retrogradation (Liu et al. 2021). These findings are consistent with previous studies on the effects of polysaccharides on starch functional properties (Ji, Yang, et al. 2021; Ji, Yin, et al. 2021; Lutfi et al. 2017). Among the tested SDFs, IN had a greater impact on CS parameters compared to RD and Sta at the same concentration. IN's longer molecular chain may allow it to form more effective coatings on the starch granule surface, inhibiting water absorption and swelling during pasting, which aligns with the results of the SP and S experiments.

### 3.3 Particle Size Changes

**FIGURE 2:**
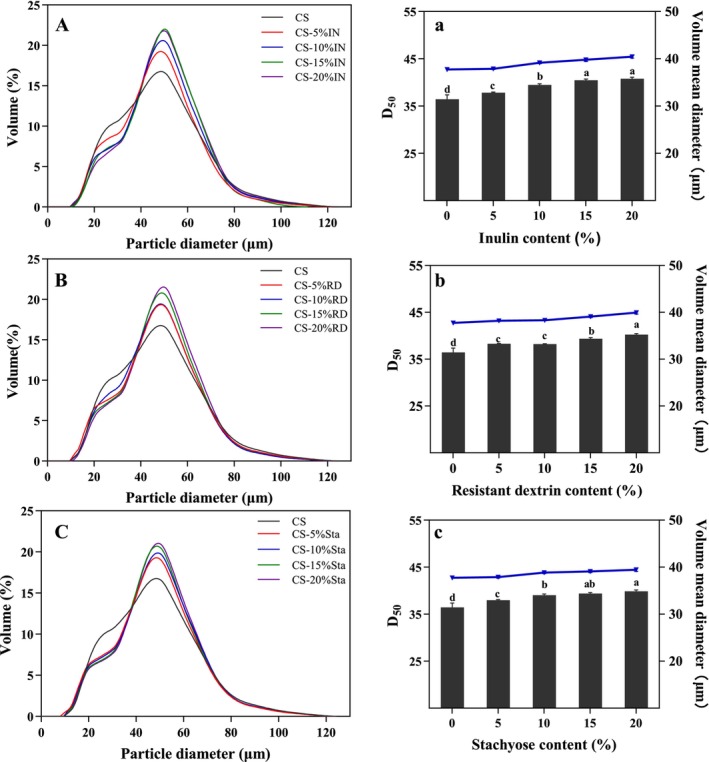
The effect of different dietary fibers at various addition levels on the particle size changes of CS and CS‐SDFs during gelatinization. Different letters indicate significant differences between the data (*p* < 0.05).

To investigate the effects of the three SDFs on the gelatinization characteristics of CS, a laser particle size analyzer was used to measure the particle size distribution of CS and CS‐SDFs mixtures after gelatinization. The *D*_50_ value, representing the median particle size, indicates the particle size at which 50% of the sample's cumulative distribution is larger and 50% is smaller (Ji, Yang, et al. 2021; Ji, Yin, et al. 2021). As shown in Figure 2, the addition of SDFs significantly increased the particle size of gelatinized starch, with both *D*_50_ and average particle size showing similar trends (*p* < 0.05). Specifically, when 20% of IN, RD, and Sta were added, the average particle size increased from 36.45 μm (native CS) to 40.80 μm, 40.27 μm, and 39.86 μm, respectively.

This increase in particle size can be attributed to the encapsulation of starch granules by SDFs during gelatinization. IN and RD had stronger effects on particle size compared to Sta, likely due to their larger molecular structures forming a more effective coating around starch granules. In contrast, Sta's smaller molecular weight and unique tetrasaccharide structure may limit its ability to form a robust coating. Similar findings have been reported by Xu et al. (2023), who observed that the addition of beet pectin increased the particle size of sweet potato starch due to its encapsulation effects. Additionally, Han et al. (2024) demonstrated that *Eucommia ulmoides* leaf powder enhanced the *D*_50_ and average particle size of sweet potato starch, consistent with our results. Under the combined effects of heating and shearing during gelatinization, the addition of SDFs enhanced the structural integrity of starch granules by forming a protective coating, which prevented rupture despite full swelling (Xu et al. 2023). This encapsulation effect of SDFs not only maintained granule stability but also contributed to the observed increase in particle size, consistent with our experimental results.

### 3.4 Digestibility Characteristics

Starch digestion is closely linked to the GI and the sustained release of starch energy. Based on digestion rate, starch is classified as rapidly digestible starch (RDS), slowly digestible starch (SDS), and resistant starch (RS) (Zhang et al. 2023). SDS and RS contribute to lower glycemic responses, reducing the risk of chronic diseases. RS, which resists amylase hydrolysis, undergoes fermentation by gut microorganisms to produce short‐chain fatty acids such as acetate, propionate, and butyrate, which can help regulate blood glucose levels (Ji, Yang, et al. 2021; Ji, Yin, et al. 2021).

**FIGURE 3:**
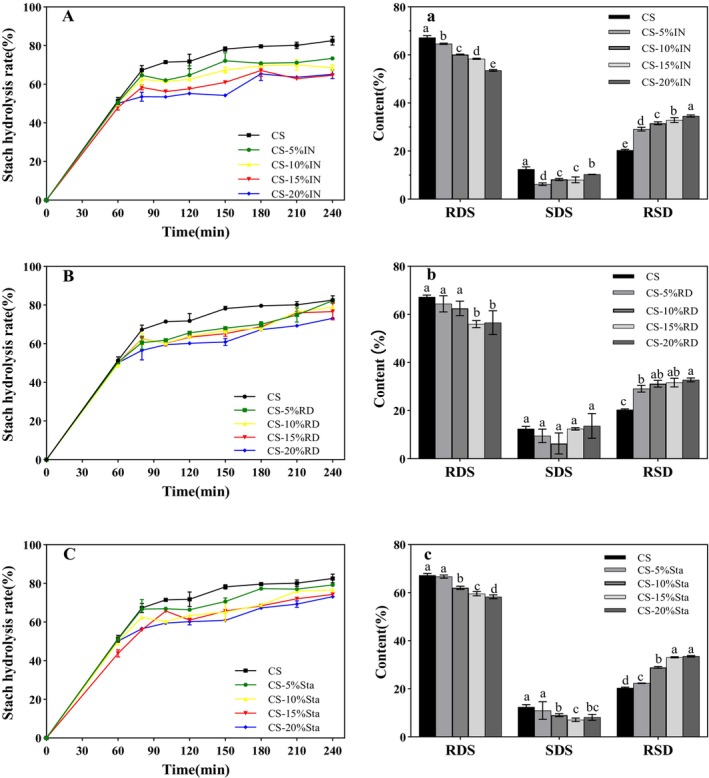
Effect of different contents of IN, RD, Sta on the starch digestibility fractions and hydrolysis of corn starch. (A, a) IN; (B, b) RD; (C, c) Sta. Different letters indicate significant differences between the data (*p* < 0.05).

The effects of IN, RD, and Sta on the in vitro digestion characteristics of gelatinized CS are presented in Figure 3. The inclusion of SDFs significantly reduced the hydrolysis rate of gelatinized CS, and this inhibitory effect became stronger with increasing SDFs levels. As shown in Figure 3, SDFs significantly decreased RDS content and increased RS content (*p* < 0.05), with both trends becoming more pronounced as SDFs concentrations increased. For instance, when the IN content reached 20%, RDS decreased from 67.25% to 55.13%, while RS increased from 20.34% to 34.60%. Similar effects were observed for RD and Sta: at 20%, RDS was reduced to 56.54% and 58.31%, while RS increased to 37.66% and 33.55%, respectively. However, SDS content exhibited different trends: IN reduced SDS significantly at concentrations ≥ 5%, while Sta showed reductions at ≥ 10%. In contrast, RD had no significant effect on SDS content (*p* > 0.05).

These results show that SDFs can reduce starch digestibility, possibly by restricting water migration during digestion. The high water‐holding capacity of SDFs limits water penetration into starch granules, reducing swelling and hydrolysis rates. Among the SDFs, IN exhibited the strongest inhibitory effect, likely due to its longer molecular chains forming effective coatings around starch molecules. This supports the hypothesis that SDFs compete with starch for water, disrupting the gelatinization process, and aligns with earlier findings on SP and pasting characteristics.

#### 3.4.1 Scanning Electron Microscopy(SEM)

**FIGURE 4:**
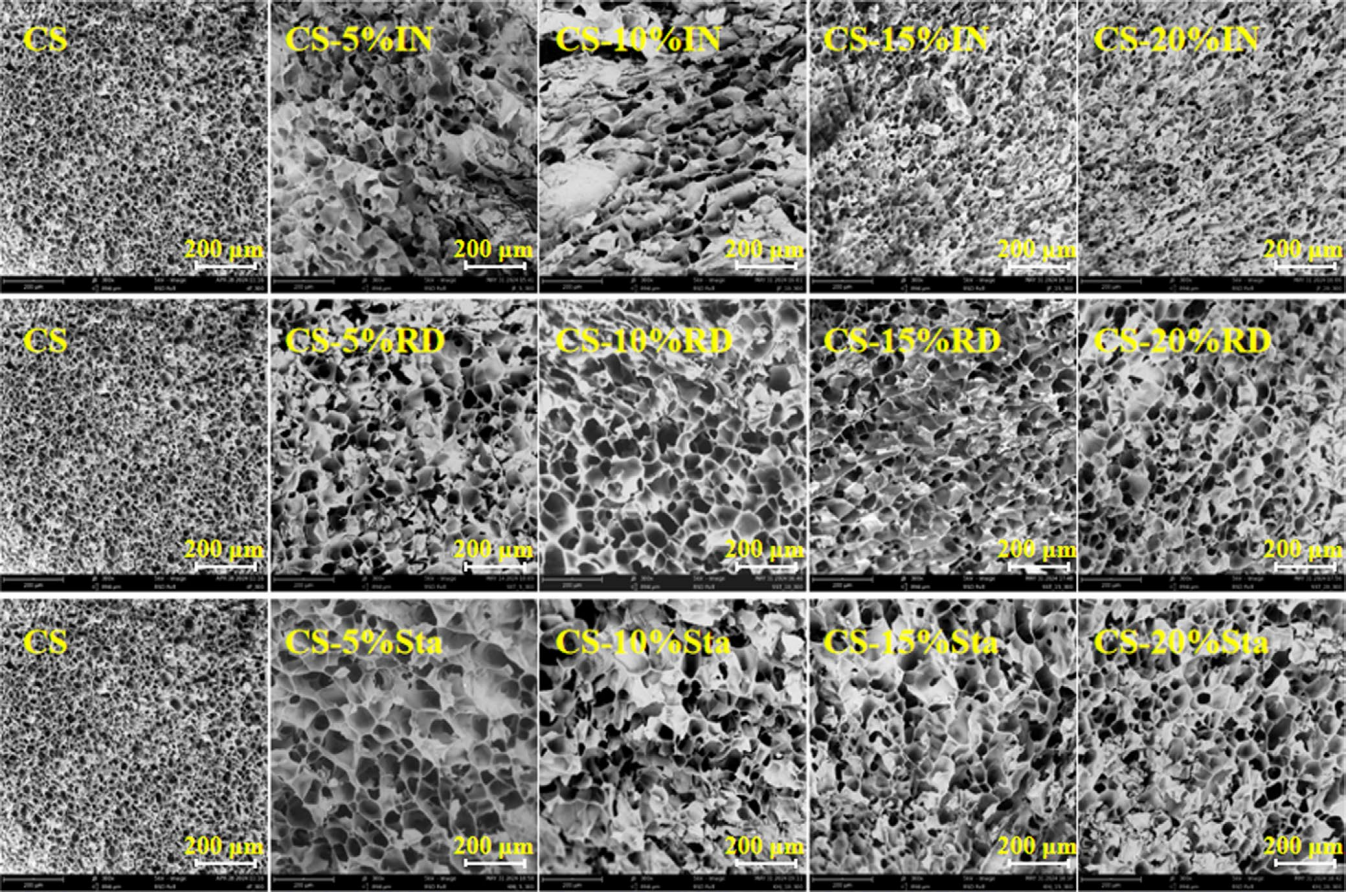
The electron microscope images (300×) of gelatinized corn starch with different levels of SDFs.

The microstructural characteristics of CS and CS‐SDFs mixtures after freeze‐drying were observed using scanning electron microscopy (SEM). The SEM images illustrate the effects of different SDFs levels on the morphological characteristics of CS gels (Figure 4). The gelatinized CS granules exhibited disrupted structures with visible pores, likely caused by ice crystal formation and thawing during freeze‐drying, which rearranged starch molecular chains and created porous structures after significant water loss (Zheng, Huang, et al. 2021; Zheng, Wang, et al. 2021). These porous structures are consistent with the findings of Liu et al. (2021) and Ren et al. (2020) regarding gelatinized and freeze‐dried starch.

The addition of SDFs significantly altered the microstructure of CS gels compared to the control group (*p* < 0.05). SDFs‐containing samples exhibited more pronounced layered structures and aggregation, likely due to the hydrophilicity and water‐binding properties of SDFs. SDFs convert free water in the starch gel system into bound water, reducing ice crystal formation and dehydration shrinkage, thereby lowering the syneresis rate of CS gels (Lan et al. 2024). Additionally, SDFs interact with amylose molecules leached during gelatinization, promoting molecular aggregation and forming a more compact structure that limits water penetration during gelatinization.

Notably, gelatinized CS with high IN concentrations exhibited smaller pore sizes and denser stacking, indicating a more compact gel structure. This may be attributed to the strong hydrophilicity of IN, which binds water more effectively and prevents its separation from the starch matrix (Ji, Yang, et al. 2021; Ji, Yin, et al. 2021). Similar phenomena of reduced pore size in starch gels with high polysaccharide concentrations have been reported by Jia et al. (2023) and Song et al. (2024). These results suggest that SDFs enhance the formation of a robust three‐dimensional network with CS, resulting in denser gel structures.

### 3.5 FT‐IR Spectrum Analysis

**FIGURE 5:**
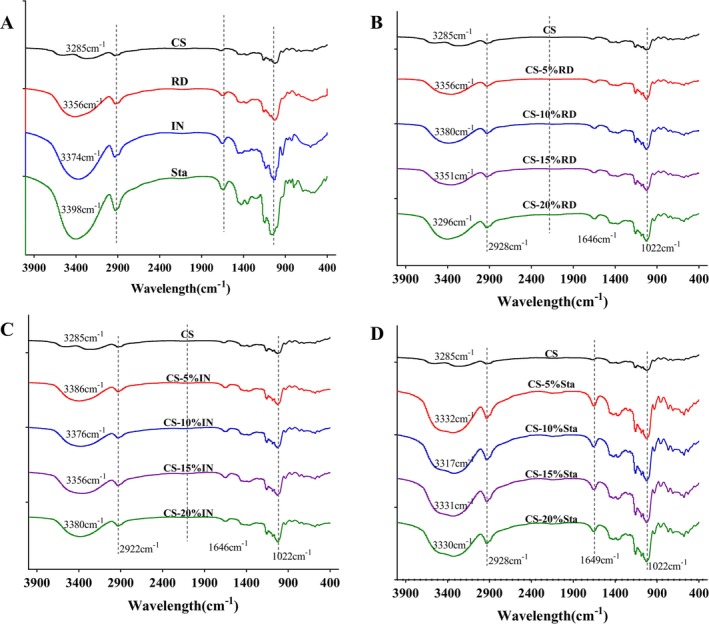
The FT‐IR variations of different samples (A) corn starch and three different SDFs, (B) corn starch with varying levels of IN, (C) corn starch with varying levels of RD, (D) corn starch with varying levels of Sta).

In food systems, dietary fibers interact with starch through electrostatic forces, hydrogen bonding, hydrophobic interactions, and van der Waals forces (Ren et al. 2020). As shown in Figure 5A, the FT‐IR spectra of gelatinized CS and SDFs (IN, RD, and Sta) reveal the presence of numerous hydrophilic groups, particularly hydroxyl groups, indicating that hydrogen bonds and electrostatic interactions are the primary forces driving their interactions (Ren et al. 2020). The FT‐IR absorption spectra of CS, CS‐IN, CS‐RD, and CS‐Sta mixtures (4000–400 cm^−1^) are shown in Figure 5B–D. No new absorption peaks appeared after the addition of SDFs, suggesting that the interactions between CS and SDFs occur through non‐covalent bonds. The broadening and intensification of the ‐OH stretching peak around 3400 cm^−1^ in all SDFs‐containing samples indicate the contribution of ‐OH groups from SDFs. Notably, in the CS‐Sta mixture, the –OH peak intensity decreased as Sta concentration increased, likely due to its smaller molecular weight and higher hydroxyl content, which allow Sta to penetrate starch molecules more effectively and interact directly with them (Jia et al. 2023).

The 1047/1022 cm^−1^ and 995/1022 cm^−1^ absorbance ratios of the samples (Table 2) further illustrate the structural changes in CS. The 1047/1022 cm^−1^ ratio reflects the short‐range order degree (DO), while the 995/1022 cm^−1^ ratio represents the degree of double helix structure (DD) (Zhou et al. 2021; Lan et al. 2024). As shown in Table 2, the DO and DD values of CS significantly increased after the addition of IN, RD, and Sta, suggesting that SDFs enhance starch aggregation during gelatinization via hydrogen bonding and electrostatic interactions (Gao et al. 2021). Among the SDFs, Sta had the greatest impact on DO and DD values, with maximum increases of 9.15% and 96.01%, compared to 3.58% and 18.21% for IN and 3.42% and 25.39% for RD. This difference may be due to Sta's smaller molecular weight and higher hydroxyl content, allowing it to penetrate starch molecules more effectively, consistent with the FT‐IR results.

**TABLE 2:** DO and DD values of different samples.

Sample	DO	DD
CS	1.0204 ± 0.0003^i^	1.1723 ± 0.0008^j^
CS‐5%IN	1.0407 ± 0.0007^gh^	1.3097 ± 0.0009^i^
CS‐10%IN	1.0390 ± 0.0009^h^	1.3173 ± 0.0006^h^
CS‐15%IN	1.0428 ± 0.0007^g^	1.3644 ± 0.0009^g^
CS‐20%IN	1.0570 ± 0.0010^d^	1.3858 ± 0.0006^f^
CS‐5%RD	1.0471 ± 0.0008^a^	1.3132 ± 0.0009^hi^
CS‐10%RD	1.0524 ± 0.0009^a^	1.3870 ± 0.0012^f^
CS‐15%RD	1.0418 ± 0.0007^b^	1.3110 ± 0.0008^hi^
CS‐20%RD	1.0553 ± 0.0011^c^	1.4699 ± 0.0011^e^
CS‐5%Sta	1.1139 ± 0.0016^f^	2.2941 ± 0.0069^a^
CS‐10%Sta	1.1136 ± 0.0015^e^	2.2116 ± 0.0065^b^
CS‐15%Sta	1.1087 ± 0.0015^g^	2.1321 ± 0.0054^c^
CS‐20%Sta	1.0975 ± 0.0012^d^	1.9188 ± 0.0044^d^

*Note:* Reported values correspond to the mean ± standard deviation. Different letters in the same column indicate significant differences (*p* < 0.05).

In conclusion, the addition of SDFs strengthens the ordered and double helix structures of CS after gelatinization, with Sta showing the most significant impact due to its molecular characteristics.

### 3.6 Glucose Adsorption Capacity of SDFs

**FIGURE 6:**
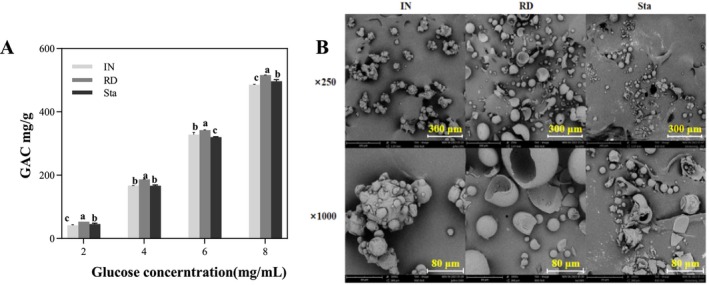
The glucose adsorption capacity (A) and electron microscopy images (B) of different SDFs.

The glucose adsorption capacity reflects the glucose‐binding behavior of SDFs during gastrointestinal transit in vitro. As shown in Figure 6A, the glucose adsorption capacity of IN, RD, and Sta increased with rising glucose concentrations, reaching maximum values of 485.66 mg g^−1^, 516.15 mg g^−1^, and 496.88 mg g^−1^, respectively. RD exhibited a significantly higher glucose adsorption capacity than IN and Sta (*p* < 0.05). These results are consistent with previous studies showing that dietary fibers from various sources absorb glucose in a dose‐dependent manner (Dong et al. 2023).

To explore the structural differences underlying glucose adsorption, the morphological characteristics of IN, RD, and Sta were observed via electron microscopy (Figure 6B). IN displayed a spherical structure with surface wrinkles and intact particles, resulting in lower porosity and reduced glucose adsorption. In contrast, RD exhibited irregular particle sizes, smooth surfaces, and hollow interiors caused by enzymatic and thermal processing, which enhanced porosity and provided a larger adsorption surface (Tang et al. 2022). Similarly, Sta showed hollow interiors and wrinkled surfaces but smaller particle sizes, which limited its adsorption capacity compared to RD. These observations align with previous studies suggesting that porosity and surface structure play key roles in glucose adsorption (Dong et al. 2023; Hu et al. 2022; Tang et al. 2022; Li et al. 2022).

Compared to IN, RD exhibits superior glucose adsorption capacity, which can be attributed to its higher porosity and incomplete particle structure, providing a larger surface area for glucose binding (Huo et al. 2024; Zheng, Wang, et al. 2022; Zheng, Xu, et al. 2022). Similarly, RD outperforms Sta, likely due to its longer molecular chains that facilitate the capture of glucose molecules within the fiber network (Tang et al. 2022). These findings suggest that differences in the structural properties and molecular weights of SDFs contribute to their varying glucose adsorption capacities, which may delay glucose diffusion in the gastrointestinal tract and suppress postprandial blood glucose spikes.

### 3.7 Interaction of Three Different SDFs With α‐Amylase

#### 3.7.1 Inhibition of α‐Amylase

**FIGURE 7:**
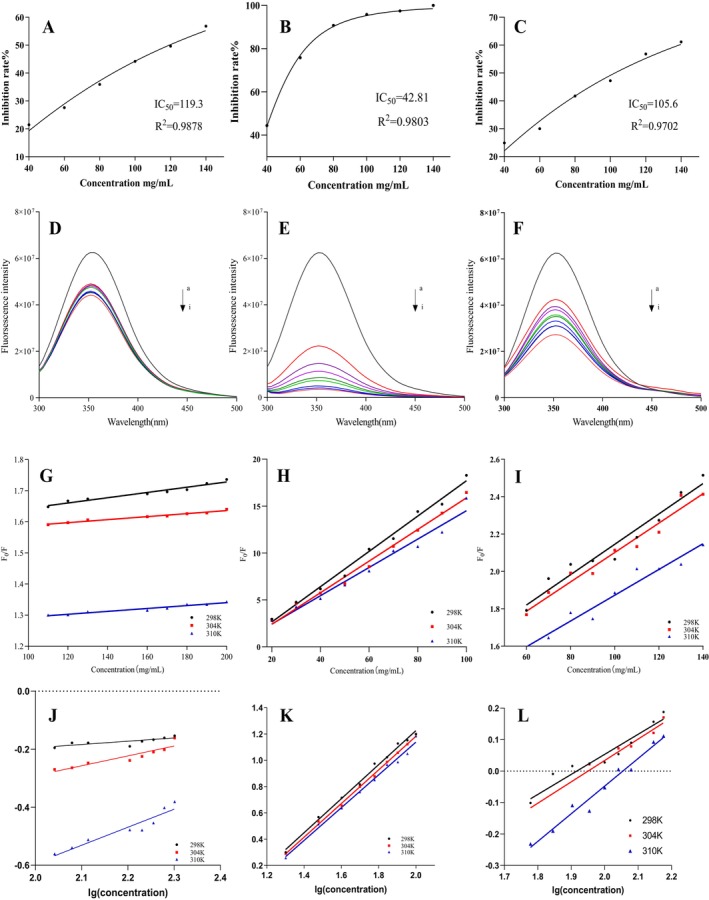
Inhibitory activity of IN, RD, and Sta on α‐amylase (A) IN, (B) RD, (C) Sta. Fluorescence spectra of the interaction between α‐amylase and IN, RD, Sta (D) IN, (E) RD, (F) Sta, along with fluorescence quenching curves at different temperatures (298 K, 304 K, 310 K) (G, J) IN, (H, K) RD, (I, L) Sta.

The α‐amylase and α‐glucosidase enzymes are key mediators of carbohydrate digestion in mammals, responsible for breaking down carbohydrates into absorbable forms. The experimental results showed that at a concentration of 300 mg mL^−1^, none of the three SDFs exhibited significant inhibitory effects on α‐glucosidase. Therefore, this study focused on the inhibitory effects of SDFs on α‐amylase, as shown in Figure 7A–C. The IC_50_ values of RD, IN, and Sta for α‐amylase were 42.81 mg mL^−1^, 119.3 mg mL^−1^, and 105.6 mg mL^−1^, respectively, indicating that RD exhibited the strongest α‐amylase inhibitory effect, followed by Sta and IN.

The superior inhibitory effect of RD on α‐amylase can be attributed to its microstructure. The greater degree of damage to RD particles during processing increases the contact area and binding sites available for α‐amylase, enhancing its binding capacity and reducing enzyme‐starch interactions (Chen et al. 2021; Wang et al. 2021). Zheng, Huang, et al. (2021) and Zheng, Wang, et al. (2021) reported that the porous structure formed during the modification of wheat bran dietary fibers improved its α‐amylase inhibitory activity, consistent with the structural features of RD. Sta also showed a stronger inhibitory effect on α‐amylase compared to IN, which may result from its higher hydroxyl group content at the same addition level. Studies have shown that stronger hydrophilic hydroxyl groups can enhance hydrogen bonding between dietary fibers and α‐amylase active sites, reducing enzymatic activity (Zheng, Huang, et al. 2021; Zheng, Wang, et al. 2021).

In summary, the α‐amylase inhibitory effects of SDFs are closely related to their structural characteristics, including porosity, molecular chain length, and hydroxyl group content. Among the three SDFs, RD demonstrated the strongest inhibitory activity, highlighting its potential for use in functional foods aimed at glycemic control.

#### 3.7.2 Fluorescence Spectroscopy

To further investigate the impact of SDFs on the structure of α‐amylase, fluorescence spectroscopy was used to observe changes in α‐amylase before and after the addition of SDFs. As shown in Figure 5D–F, α‐amylase exhibits strong fluorescence at 352 nm, primarily due to the intrinsic fluorescence of aromatic amino acids such as tyrosine (Tyr), tryptophan (Trp), and phenylalanine (Phe). The fluorescence intensity reflects the interactions between chromophore groups and surrounding groups on the enzyme surface (He et al. 2023; Sun et al. 2024). After adding IN, RD, and Sta at different concentrations, all three SDFs induced fluorescence quenching in α‐amylase, with the fluorescence intensity decreasing as SDFs concentrations increased. This quenching is likely caused by the formation of SDFs‐α‐amylase complexes, which alter the fluorescent properties of the enzyme's chromophore groups (Li et al. 2023). Notably, RD induced the most significant fluorescence quenching, consistent with its stronger inhibitory effect on α‐amylase observed in enzyme inhibition experiments, suggesting that RD forms complexes with the enzyme more efficiently.

Fluorescence quenching can occur via static quenching (formation of quencher‐fluorophore complexes), dynamic quenching (collisions between quencher and fluorophore), or a combination of both. To determine the quenching mechanism, the Stern–Volmer equation was applied to analyze fluorescence data. The quenching constant (*K*_sv_) distinguishes between static and dynamic quenching: in dynamic quenching, increasing temperature enhances molecular collisions and raises the *K*_sv_, while in static quenching, higher temperatures destabilize complexes, leading to a lower *K*_sv_. As shown in Figure 7G–L and Table 3, the quenching curves exhibit good linearity at 298 K, 304 K, and 310 K, indicating a single quenching mechanism (Sun et al. 2024). The *K*_sv_ values decreased with increasing temperature, confirming that the quenching mechanism between SDFs and α‐amylase is static.

**TABLE 3:** Quenching constants (*K*_sv_) and quenching rate constants (Kq) for the interaction of IN, RD, and Sta with α‐amylase at different temperatures (298 K, 304 K, 310 K).

Sample	*T*/*K*	*K*_sv_/(×10^−2^ L·mol^−1^)	*K*_q_ (×10^5^ L·mol^−1^·s^−1^)	*R* ^2^
IN	298	0.0137	0.0137	0.9783
	304	0.0079	0.0079	0.9838
	310	0.0076	0.0076	0.9698
RD	298	9.41	9.41	0.9516
	304	8.43	8.43	0.9641
	310	7.55	7.55	0.9564
Sta	298	1.22	1.22	0.9528
	304	1.18	1.18	0.9738
	310	1.04	1.04	0.9738

### 3.8 Analysis of the In Vitro Hypoglycemic Mechanism of Three Types of SDFs

**FIGURE 8:**
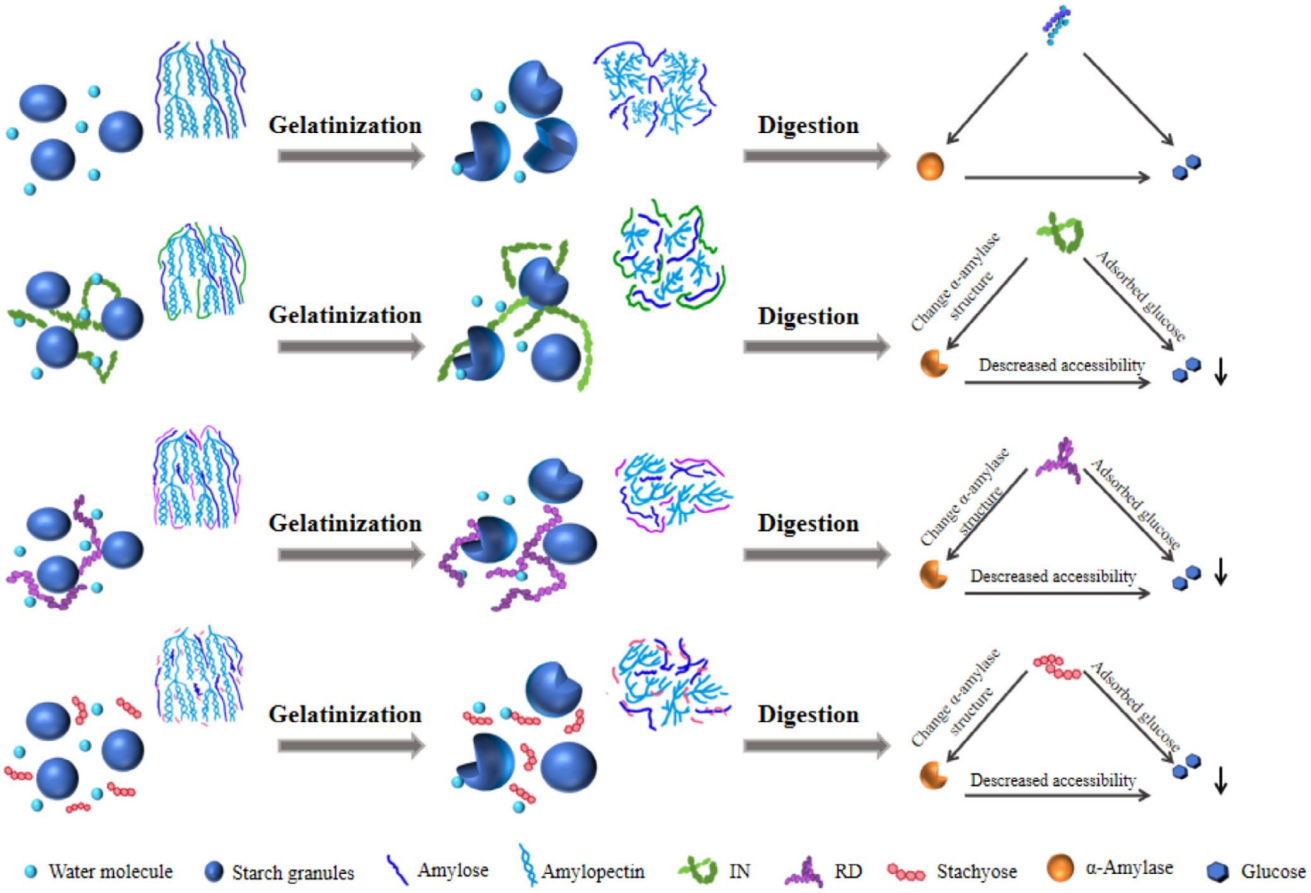
Schematic diagram of the in vitro glucose‐lowering mechanisms of IN, RD, and Sta.

Based on the experimental results, the in vitro hypoglycemic mechanisms of SDFs primarily involve four pathways: (1) encapsulating starch to hinder its contact with enzymes and water during digestion (Kong et al. 2020); (2) modifying starch structure through non‐covalent interactions, such as hydrogen bonding, to increase molecular orderliness (Zhang et al. 2021); (3) inhibiting enzyme activity or altering enzyme structure (Zheng, Wang, et al. 2022; Zheng, Xu, et al. 2022); and (4) adsorbing glucose (Zheng, Wang, et al. 2022; Zheng, Xu, et al. 2022). A schematic diagram illustrating these mechanisms, differentiated by the structural properties of the three dietary fibers, is shown in Figure 8.

IN, a linear fructan polysaccharide, exhibits high water‐holding capacity, gel‐forming ability, and low viscosity. When mixed with starch, IN competes for water, limiting water availability and migration, which raises the gelatinization temperature (Ji, Yang, et al. 2021; Ji, Yin, et al. 2021). Furthermore, IN encapsulates starch molecules, forming a three‐dimensional network that reduces starch swelling during gelatinization and decreases amylose leaching (Luo et al. 2017). RD, also a linear polysaccharide, shares similar effects on starch structure. However, RD's structural damage during high‐temperature enzymatic hydrolysis increases its surface area and porosity, providing more binding sites for glucose molecules and enhancing its adsorption capacity (Zheng, Huang, et al. 2021; Zheng, Wang, et al. 2021). Additionally, RD also exhibited strong α‐amylase inhibitory activity. Sta, with its unique tetrasaccharide structure, more easily inserts itself into starch molecules during heating. Its higher hydroxyl group content facilitates stronger hydrogen bonding with amylose chains, promoting the formation of a more ordered spatial structure and increasing the crystallinity of starch molecules (Zheng, Huang, et al. 2021; Zheng, Wang, et al. 2021). These structural properties allow Sta to strengthen starch's resistance to digestion.

## 4 Conclusion

This study investigated the mechanisms by which three SDFs—IN, RD, and Sta—delay the in vitro digestion of CS. The focus was on their effects on the SP, S, pasting properties, structure, and in vitro hydrolysis rate of CS, as well as their α‐amylase inhibitory mechanisms and glucose adsorption capacity. The results demonstrated that IN reduces starch digestion by encapsulating starch granules and competing for water, thereby limiting starch swelling and amylose leaching. RD exhibited strong glucose adsorption capacity and α‐amylase inhibitory activity. Sta, with its high hydroxyl group content, penetrated the starch molecules, enhanced molecular orderliness and double‐helical structure, and reduced starch digestibility. These findings indicate that SDFs can significantly modify the digestion and absorption of starch‐based foods, offering valuable strategies for lowering the glycemic index and enriching dietary fiber content in functional foods.

## Conflicts of Interest

The authors declare no conflicts of interest.

## Supporting information


Data S1.

## Data Availability

Data are made available upon reasonable request by contacting the corresponding author.

## References

[fsn370165-bib-0001] Chen, H. , M. Xiong , T. Bai , et al. 2021. “Comparative Study on the Structure, Physicochemical, and Functional Properties of Dietary Fiber Extracts From Quinoa and Wheat.” LWT 149: 111–816. 10.1016/j.lwt.2021.111816.

[fsn370165-bib-0002] Dong, Y. , Q. Li , Y. Guo , Y. Zhao , and J. Cao . 2023. “Comparison of Physicochemical and In Vitro Hypoglycemic Activity of Bamboo Shoot Dietary Fibers From Different Regions of Yunnan.” Frontiers in Nutrition 9, no. 1: 102671. 10.3389/fnut.2022.1102671. PMC9879356 36712536

[fsn370165-bib-0003] Englyst, H. N. , S. M. Kingman , and J. H. Cummings . 1992. “Classification and Measurement of Nutritionally Important Starch Fractions.” European Journal of Clinical Nutrition 46: S33–S50. 1330528

[fsn370165-bib-0004] Gao, J. , L. Zhu , J. Huang , et al. 2021. “Effect of Dandelion Root Polysaccharide on the Pasting, Gelatinization, Rheology, Structural Properties and In Vitro Digestibility of Corn Starch.” Food & Function 12, no. 15: 7029–7039. 10.1039/D1FO00507C. 34152329

[fsn370165-bib-0005] Han, S. , Y. Wu , S. Chen , et al. 2024. “Effects of Eucommia Ulmoides Leaf Powder on the Gelatinization, Rheology, and Gel Properties of Sweet Potato Starch.” LWT 203: 116–428.

[fsn370165-bib-0006] He, T. , X. Zhang , L. Zhao , et al. 2023. “Insoluble Dietary Fiber From Wheat Bran Retards Starch Digestion by Reducing the Activity of Alpha‐Amylase.” Food Chemistry 426: 136–624. 10.1016/j.foodchem.2023.136624. 37356242

[fsn370165-bib-0007] Hu, K. , D. Chen , and Z. Sun . 2022. “Structures, Physicochemical Properties, and Hypoglycemic Activities of Soluble Dietary Fibers From White and Black Glutinous Rice Bran: A Comparative Study.” Food Research International 159: 111–423. 10.1016/j.foodres.2022.111423. 35940748

[fsn370165-bib-0008] Huo, Y. , Y. Niu , S. Feng , L. Zhang , J. Xu , and Q. Hu . 2024. “Effect of Three Modification Methods on Physical and Functional Properties of Flaxseed Cake Dietary Fiber.” LWT 198: 116076. 10.1016/j.lwt.2024.116076.

[fsn370165-bib-0009] Ji, H. , P. Yang , L. Zhang , et al. 2021. “Effects of Inulin With Short and Long‐Chain on Pasting, Texture and Rheological Properties of Sweet Potato Starch.” CyTA Journal of Food 19, no. 1: 21–32. 10.1080/19476337.2020.1852314.

[fsn370165-bib-0010] Ji, X. , M. Yin , L. Hao , M. Shi , H. Liu , and Y. Liu . 2021. “Effect of Inulin on Pasting, Thermal, Rheological Properties and In Vitro Digestibility of Pea Starch Gel.” International Journal of Biological Macromolecules 193: 1669–1675. 10.1016/j.ijbiomac.2021.11.004. 34742552

[fsn370165-bib-0011] Jia, S. , H. Tao , H. Zhao , et al. 2023. “Effects of Lycium Barbarum Polysaccharide on Pasting, Rheology, and In Vitro Digestibility of Starch.” Starch—Stärke 75, no. 1–2: 2. 10.1002/star.202200185.

[fsn370165-bib-0012] Kong, X.‐R. , Z.‐Y. Zhu , X.‐J. Zhang , and Y.‐M. Zhu . 2020. “Effects of Cordyceps Polysaccharides on Pasting Properties and In Vitro Starch Digestibility of Wheat Starch.” Food Hydrocolloids 102: 105–604. 10.1016/j.foodhyd.2019.105604.

[fsn370165-bib-0013] Lan, G. , S. Xie , Q. Duan , et al. 2024. “Effect of Soybean Polysaccharide and Soybean Oil on Gelatinization and Retrogradation Properties of Corn Starch.” International Journal of Biological Macromolecules 264: 130–772. 10.1016/j.ijbiomac.2024.130772. 38467217

[fsn370165-bib-0014] Li, J. , J. Zhang , W. Yu , et al. 2023. “Soluble Dietary Fibres Decrease α‐Glucosidase Inhibition of Epigallocatechin Gallate Through Affecting Polyphenol‐Enzyme Binding Interactions.” Food Chemistry 409: 135327. 10.1016/j.foodchem.2022.135327. 36586254

[fsn370165-bib-0015] Li, L. , J. Liu , Y. Zhang , Q. Wang , and J. Wang . 2022. “Qualitative and Quantitative Correlation of Microstructural Properties and In Vitro Glucose Adsorption and Diffusion Behaviors of Pea Insoluble Dietary Fiber Induced by Ultrafine Grinding.” Food 11, no. 18: 2814. 10.3390/foods11182814. PMC9497999 36140942

[fsn370165-bib-0016] Liu, C. , H. Zhang , R. Chen , et al. 2021. “Effects of Creeping Fig Seed Polysaccharide on Pasting, Rheological, Textural Properties and In Vitro Digestibility of Potato Starch.” Food Hydrocolloids 118: 106810. 10.1016/j.foodhyd.2021.106810.

[fsn370165-bib-0017] Luo, D. , Y. Li , B. Xu , et al. 2017. “Effects of Inulin With Different Degree of Polymerization on Gelatinization and Retrogradation of Wheat Starch.” Food Chemistry 229: 35–43. 10.1016/j.foodchem.2017.02.058. 28372184

[fsn370165-bib-0018] Lutfi, Z. , A. Nawab , F. Alam , A. Hasnain , and S. Z. Haider . 2017. “Influence of Xanthan, Guar, CMC and Gum Acacia on Functional Properties of Water Chestnut (Trapa Bispinosa) Starch.” International Journal of Biological Macromolecules 103: 220–225. 10.1016/j.ijbiomac.2017.05.046. 28506726

[fsn370165-bib-0019] Ma, S. , P. Zhu , and M. Wang . 2019. “Effects of Konjac Glucomannan on Pasting and Rheological Properties of Corn Starch.” Food Hydrocolloids 89: 234–240. 10.1016/j.foodhyd.2018.10.045.

[fsn370165-bib-0020] Qin, X.‐Y. , J.‐T. Zhang , G.‐M. Li , et al. 2020. “Structure and Composition of a Potential Antioxidant Obtained From the Chelation of Pea Oligopeptide and Sodium Selenite.” Journal of Functional Foods 64: 103–619. 10.1016/j.jff.2019.103619.

[fsn370165-bib-0021] Qiu, S. , M. P. Yadav , Y. Liu , H. Chen , E. Tatsumi , and L. Yin . 2016. “Effects of Corn Fiber Gum With Different Molecular Weights on the Gelatinization Behaviors of Corn and Wheat Starch.” Food Hydrocolloids 53: 180–186. 10.1016/j.foodhyd.2015.01.034.

[fsn370165-bib-0022] Ren, Y. , L. Jiang , W. Wang , et al. 2020. “Effects of Mesona Chinensis Benth Polysaccharide on Physicochemical and Rheological Properties of Sweet Potato Starch and Its Interactions.” Food Hydrocolloids 99: 105–371. 10.1016/j.foodhyd.2019.105371.

[fsn370165-bib-0023] Song, J. , L. Rong , J. Li , et al. 2024. “Effects of Three Different Polysaccharides on the Sol Gel‐Behavior, Rheological, and Structural Properties of Tapioca Starch.” International Journal of Biological Macromolecules 254: 128053. 10.1016/j.ijbiomac.2023.128053. 37963504

[fsn370165-bib-0024] Sun, N. , J. Xie , B. Zheng , et al. 2024. “The Inhibition Mechanism of Bound Polyphenols Extracted From Mung Bean Coat Dietary Fiber on Porcine Pancreatic α‐Amylase: Kinetic, Spectroscopic, Differential Scanning Calorimetric and Molecular Docking.” Food Chemistry 436: 137749. 10.1016/j.foodchem.2023.137749. 37864970

[fsn370165-bib-0025] Tang, C. , L. Wu , F. Zhang , J. Kan , and J. Zheng . 2022. “Comparison of Different Extraction Methods on the Physicochemical, Structural Properties, and In Vitro Hypoglycemic Activity of Bamboo Shoot Dietary Fibers.” Food Chemistry 386: 132–642. 10.1016/j.foodchem.2022.132642. 35349899

[fsn370165-bib-0026] Wang, K. , M. Li , Q. Han , R. Fu , and Y. Ni . 2021. “Inhibition of α‐Amylase Activity by Insoluble and Soluble Dietary Fibers From Kiwifruit (Actinidia Deliciosa).” Food Bioscience 42: 101057. 10.1016/j.fbio.2021.101057.

[fsn370165-bib-0027] Wang, R. , J. Wan , C. Liu , X. Xia , and Y. Ding . 2019. “Pasting, Thermal, and Rheological Properties of Rice Starch Partially Replaced by Inulin With Different Degrees of Polymerization.” Food Hydrocolloids 92: 228–232. 10.1016/j.foodhyd.2019.02.008.

[fsn370165-bib-0028] Wang, X. , Z. Yang , S. Shen , et al. 2023. “Inhibitory Effects of Chlorophylls and Its Derivative on Starch Digestion In Vitro.” Food Chemistry 413: 135377. 10.1016/j.foodchem.2022.135377. 36773358

[fsn370165-bib-0029] Witczak, T. , M. Witczak , and R. Ziobro . 2014. “Effect of Inulin and Pectin on Rheological and Thermal Properties of Potato Starch Paste and Gel.” Journal of Food Engineering 124: 72–79. 10.1016/j.jfoodeng.2013.10.005.

[fsn370165-bib-0030] Xie, J. , L. Cheng , Z. Li , C. Li , Y. Hong , and Z. Gu . 2024. “Effect of Non‐Starch Components on the Structural Properties, Physicochemical Properties and In Vitro Digestibility of Waxy Highland Barley Starch.” International Journal of Biological Macromolecules 255: 128013. 10.1016/j.ijbiomac.2023.128013. 37951447

[fsn370165-bib-0031] Xiong, M. , B. Chen , Y. Chen , et al. 2024. “Effects of Soluble Dietary Fiber From Pomegranate Peel on the Physicochemical Properties and In‐Vitro Digestibility of Sweet Potato Starch.” International Journal of Biological Macromolecules 273: 133–141. 10.1016/j.ijbiomac.2024.133041. 38857720

[fsn370165-bib-0032] Xu, L. , J. Ren , X. Wang , Z. Bai , S. Chai , and X. Wang . 2023. “Effects of Sugar Beet Pectin on the Pasting, Rheological, Thermal, and Microstructural Properties of Wheat Starch.” International Journal of Biological Macromolecules 253: 127–328. 10.1016/j.ijbiomac.2023.127328. 37820921

[fsn370165-bib-0033] Xu, Y. , F. He , C. Jin , J. Su , and K. Ding . 2024. “Stachyose With Effect on Anti‐Angiogenic Activity From Salvia Yunnanensis.” Journal of Functional Foods 112: 105–971. 10.1016/j.jff.2023.105971.

[fsn370165-bib-0034] Ye, J. , X. Hu , S. Luo , D. J. McClements , L. Liang , and C. Liu . 2018. “Effect of Endogenous Proteins and Lipids on Starch Digestibility in Rice Flour.” Food Research International 106: 404–409. 10.1016/j.foodres.2018.01.008. 29579941

[fsn370165-bib-0035] Yu, S. , K. Dong , B. L. R. Pora , and J. Hasjim . 2022. “The Roles of a Native Starch and a Resistant Dextrin in Texture Improvement and Low Glycemic Index of Biscuits.” PRO 10, no. 11: 2404. 10.3390/pr10112404.

[fsn370165-bib-0036] Zhang, H. , Z. Li , L. Zhang , et al. 2021. “Effects of Soluble Dietary Fibers on the Viscosity Property and Digestion Kinetics of Corn Starch Digesta.” Food Chemistry 338: 127–825. 10.1016/j.foodchem.2020.127825. 32810814

[fsn370165-bib-0037] Zhang, Y. , Y. Wang , B. Yang , et al. 2023. “Effects of Zucchini Polysaccharide on Pasting, Rheology, Structural Properties and In Vitro Digestibility of Potato Starch.” International Journal of Biological Macromolecules 253: 127–177. 10.1016/j.ijbiomac.2023.127077. 37769764

[fsn370165-bib-0038] Zheng, J. , S. Huang , R. Zhao , N. Wang , J. Kan , and F. Zhang . 2021. “Effect of Four Viscous Soluble Dietary Fibers on the Physicochemical, Structural Properties, and In Vitro Digestibility of Rice Starch: A Comparison Study.” Food Chemistry 362: 130–181. 10.1016/j.foodchem.2021.130181. 34082291

[fsn370165-bib-0039] Zheng, M. , Q. You , Y. Lin , et al. 2019. “Effect of Guar Gum on the Physicochemical Properties and In Vitro Digestibility of Lotus Seed Starch.” Food Chemistry 272: 286–291. 10.1016/j.foodchem.2018.08.029. 30309546

[fsn370165-bib-0040] Zheng, Y. , P. Shi , Y. Li , Z. Yongliang , X. Wang , and L. Liu . 2020. “Effects of Carboxymethylation, Hydroxypropylation and Dual‐Enzyme Hydrolysis Combination With Heating on In Vitro Hypoglycaemic Properties of Coconut Cake Dietary Fibres.” International Journal of Food Science & Technology 55, no. 11: 3503–3514. 10.1111/ijfs.14701.

[fsn370165-bib-0041] Zheng, Y. , Q. Wang , J. Huang , et al. 2019. “Hypoglycemic Effect of Dietary Fibers From Bamboo Shoot Shell: An In Vitro and In Vivo Study.” Food and Chemical Toxicology 127: 120–126. 10.1016/j.fct.2019.03.008. 30878528

[fsn370165-bib-0042] Zheng, Y. , X. Wang , Y. Sun , et al. 2022. “Effects of Ultrafine Grinding and Cellulase Hydrolysis Separately Combined With Hydroxypropylation, Carboxymethylation and Phosphate Crosslinking on the In Vitro Hypoglycaemic and Hypolipidaemic Properties of Millet Bran Dietary Fibre.” LWT – Food Science and Technology 172: 114–210. 10.1016/j.lwt.2022.114210.

[fsn370165-bib-0043] Zheng, Y. , X. Wang , H. Tian , et al. 2021. “Effect of Four Modification Methods on Adsorption Capacities and In Vitro Hypoglycemic Properties of Millet Bran Dietary Fibre.” Food Research International 147: 110–565. 10.1016/j.foodres.2021.110565. 34399541

[fsn370165-bib-0044] Zheng, Y. , B. Xu , P. Shi , et al. 2022. “The Influences of Acetylation, Hydroxypropylation, Enzymatic Hydrolysis and Crosslinking on Improved Adsorption Capacities and In Vitro Hypoglycemic Properties of Millet Bran Dietary Fibre.” Food Chemistry 368: 130883. 10.1016/j.foodchem.2021.130883. 34438179

[fsn370165-bib-0045] Zheng, Y. , W. Yang , W. Sun , et al. 2020. “Inhibition of Porcine Pancreatic α‐Amylase Activity by Chlorogenic Acid.” Journal of Functional Foods 64: 103–587. 10.1016/j.jff.2019.103587.

[fsn370165-bib-0046] Zhou, R. , Y. Wang , Z. Wang , K. Liu , Q. Wang , and H. Bao . 2021. “Effects of Auricularia Auricula‐Judae Polysaccharide on Pasting, Gelatinization, Rheology, Structural Properties and In Vitro Digestibility of Kidney Bean Starch.” International Journal of Biological Macromolecules 191: 1105–1113. 10.1016/j.ijbiomac.2021.09.110. 34560153

[fsn370165-bib-0047] Zou, X. , X. Xu , Z. Chao , X. Jiang , L. Zheng , and B. Jiang . 2022. “Properties of Plant‐Derived Soluble Dietary Fibers for Fiber‐Enriched Foods: A Comparative Evaluation.” International Journal of Biological Macromolecules 223: 1196–1207. 10.1016/j.ijbiomac.2022.11.008. 36347374

