# Supplementary material for: In Vitro Effects of Inulin, Resistant Dextrin, and Stachyose on the Functional Properties and Digestibility of Corn Starch
Source: Food Sci Nutr. 2025 Sep 18;13(9):e70165. doi: 10.1002/fsn3.70165 (PMC12445126; doi:10.1002/fsn3.70165)
Supplement: Supplementary file 1 — Data S1. [file FSN3-13-e70165-s001.docx]

B

A


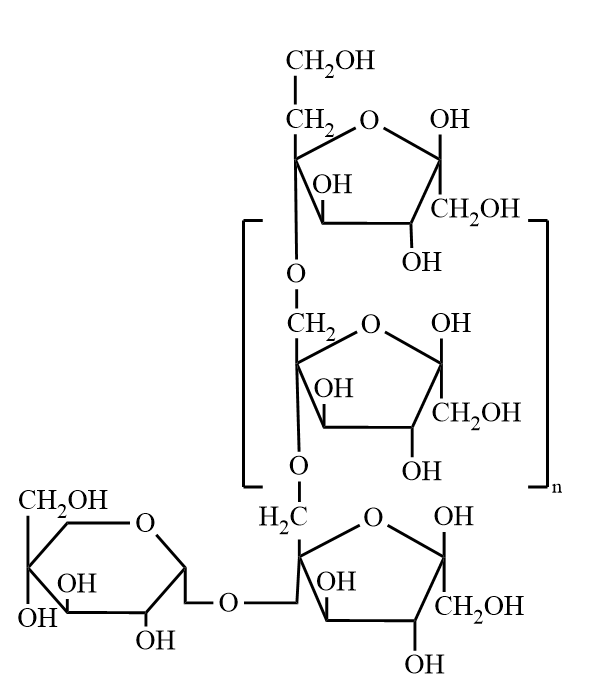

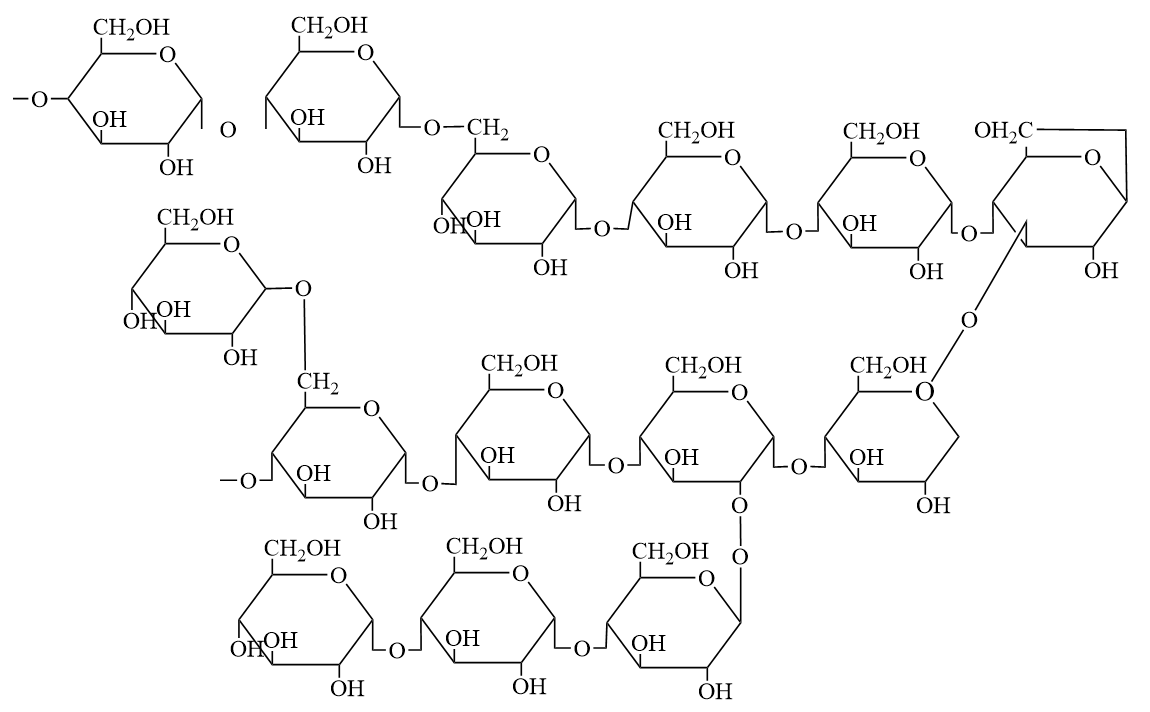


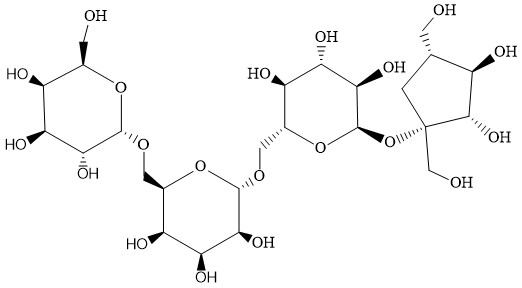


C

Fig. S_1_ Structural diagrams of IN (A), RD (B), and Sta (C).

Fig. S_2_ The effect of adding different SDFs on starch hydrolysis rate
